# Theory of Mind Is Not Theory of Emotion: A Cautionary Note on the Reading the Mind in the Eyes Test

**DOI:** 10.1037/abn0000182

**Published:** 2016-08

**Authors:** Beth F. M. Oakley, Rebecca Brewer, Geoffrey Bird, Caroline Catmur

**Affiliations:** 1 Department of Psychology, University of Cambridge, and School of Psychology, University of Surrey; 2 Medical Research Council Social, Genetic, & Developmental Psychiatry Centre, King’s College London, and School of Psychology, University of East London; 3 Medical Research Council Social, Genetic, & Developmental Psychiatry Centre, King’s College London, and Institute of Cognitive Neuroscience, University College London; 4 Department of Psychology, King’s College London, and School of Psychology, University of Surrey

**Keywords:** autism spectrum disorder, alexithymia, emotion recognition, social cognition, theory of mind

## Abstract

The ability to represent mental states (theory of mind [ToM]) is crucial in understanding individual differences in social ability and social impairments evident in conditions such as autism spectrum disorder (ASD). The Reading the Mind in the Eyes Test (RMET) is a popular measure of ToM ability, validated in part by the poor performance of those with ASD. However, the RMET requires recognition of facial emotion, which is impaired in those with alexithymia, which frequently co-occurs with ASD. Thus, it is unclear whether the RMET indexes emotion recognition, associated with alexithymia, or ToM, associated with ASD. We therefore investigated the independent contributions of ASD and alexithymia to performance on the RMET. ASD and alexithymia-matched control participants did not differ on RMET performance, whereas ASD participants demonstrated impaired performance on an alternative test of ToM, the Movie for Assessment of Social Cognition (MASC). Furthermore, alexithymia, but not ASD diagnosis, significantly influenced RMET performance but did not affect MASC performance. These results suggest that the RMET measures emotion recognition rather than ToM ability and support the alexithymia hypothesis of emotion-related deficits in ASD.

## General Scientific Summary

This study suggests that a highly popular test of the ability to detect what someone else is thinking—the Reading the Mind in the Eyes Test—is instead a test of the ability to recognize another person’s emotional expression. This is important because it suggests that patients who perform badly on this test may still be able to understand another person’s mental state and that, conversely, patients who perform well on this test may still have difficulties in mental state understanding.

Questions concerning the representation of mental states (theory of mind [ToM]) have occupied cognitive scientists for decades. The study of ToM has been particularly important in understanding individual differences in social ability and the social impairments evident in various psychiatric disorders (Baron-Cohen, Leslie, & Frith, 1985; Corcoran, Mercer, & Frith, 1995). The difficulty associated with designing a test appropriate for detecting variance in ToM ability among typical adults prompted the development of the Reading the Mind in the Eyes Test (RMET; Baron-Cohen, Wheelwright, Hill, Raste, & Plumb, 2001), which requires participants to match emotion and mental state descriptor words to images of the eye region of faces. This test has been cited over 2,000 times (Web of Science, May 2016) and has been used to demonstrate gender, cultural, genetic, and personality trait influences on ToM and elucidate its neurobiological mechanisms (Adams et al., 2010; Stone, Baron-Cohen, Calder, Keane, & Young, 2003). Its validity is supported by the poor performance of individuals with autism spectrum disorder (ASD) on the task (Baron-Cohen, Wheelwright, Hill, et al., 2001), a group that has known ToM impairments (Castelli, Frith, Happé, & Frith, 2002).

The RMET is unusual among ToM tasks in that it includes emotional states and relies on the detection of subtle facial cues, features typically used to test emotion recognition. In contrast, the majority of ToM tasks require nonemotional mental states to be inferred from contextual information or dynamic behavioral cues (Baron-Cohen, O’Riordan, Stone, Jones, & Plaisted, 1999; Dziobek et al., 2006). The reliance of the RMET on the recognition of emotional states from faces calls into question its use as a test of ToM and means that it is possible that the RMET may instead assess emotion recognition. Importantly, if the RMET does not index ToM but rather emotion recognition, then our conclusions about the ToM abilities of various cultural and psychiatric groups will require substantial revision.

It is noteworthy, therefore, that recent evidence suggests that apparent emotion recognition impairments in ASD are in fact due to alexithymia (a trait characterized by poor recognition of one’s own emotions; Nemiah, Freyberger, & Sifneos, 1976), which frequently co-occurs with ASD (the alexithymia hypothesis of emotion deficits in ASD; Bird & Cook, 2013; Cook, Brewer, Shah, & Bird, 2013). Alexithymia has been shown to predict performance on other tasks that require participants to match emotion words to faces (Grynberg et al., 2012), meaning that increased rates of alexithymia in ASD may explain the ASD impairment on the RMET if emotion recognition ability, rather than ToM, drives performance. We therefore investigated the independent contributions of ASD and alexithymia to RMET performance compared to another ToM task that does not rely exclusively on facial emotion recognition (the Movie for the Assessment of Social Cognition [MASC]; Dziobek et al., 2006). If the RMET indexes ToM, then ASD diagnostic status should predict both RMET and MASC scores. If the RMET indexes emotion recognition, however, then alexithymia, not ASD, should predict RMET performance.

## Method

### Participants

Nineteen participants (five female) with a clinical diagnosis of ASD and 24 (11 female) without ASD volunteered to take part in the study. One ASD participant had a comorbid diagnosis of dyspraxia, and one had previously been prescribed antipsychotic medication. Excluding these two participants did not alter the pattern of significance. Control participants had no past or present clinical diagnosis. One control participant did not complete the MASC, leaving the final sample at 42 participants. Based on the effect size (*d* = 1.27) between ASD and control participants on the RMET reported in Baron-Cohen, Wheelwright, Hill, et al. (2001), we determined that a sample size of 19 participants per group would provide a power of 0.8 to detect group differences using an independent *t* test and α < .05. Additional control participants were recruited to ensure that groups were matched on demographic variables. Six ASD and eight control participants met the criterion for severe alexithymia, with a score of 61 or above on the 20-item Toronto Alexithymia Scale (TAS–20; Bagby, Parker, & Taylor, 1994). The TAS–20 is a self-report scale that includes statements like *I have feelings that I cannot quite identify* and *I find it hard to describe how I feel about people.* Items are rated on a scale from 1 (*does not describe me*) to 5 (*describes me very well*), with scores ranging between 20 and 100 and higher scores indicating more alexithymic traits. The TAS–20 has good internal consistency (α = .81) and good test–retest reliability (*r* = .77; Bagby et al., 1994). The high alexithymia cutoff score of ≥61 was established as being 1.5 *SD* from the mean score of community samples (Parker, Taylor, & Bagby, 2003).

ASD and control groups were matched according to alexithymia severity, control *M* = 52.43, *SD* = 13.98; ASD *M* = 57.58, *SD* = 11.51; *t*(40) = 1.28, *p* = .207, *d* = 0.398, 95% confidence interval (CI) for *d* [−0.218, 1.009]; age, control *M* = 30.13, *SD* = 12.21; ASD *M* = 30.89, *SD* = 11.86; *t*(40) = 0.21, *p* = .839, *d* = 0.064, 95% CI for *d* [−0.545, 0.671]; gender, χ^2^(1) = 2.04, *p* = .153, *r* = .220; and IQ, measured by the Vocabulary and Matrix Reasoning subscales of the Wechsler Abbreviated Scale of Intelligence (WASI; Wechsler, 1999). These scores were as follows: control *M* = 108.48, *SD* = 11.68; ASD *M* = 109.79, *SD* = 15.71; *t*(40) = 0.31, *p* = .758, *d* = 0.096, 95% CI for *d* [−0.512, 0.704]. As shown by the IQ scores, both the ASD and control groups were in the average range for intelligence.

ASD symptom severity for all participants was measured using the Autism Spectrum Quotient (AQ; Baron-Cohen, Wheelwright, Skinner, Martin, & Clubley, 2001). The AQ is a 50-item self-report questionnaire for assessing traits associated with the autism spectrum, with statements like *I notice patterns in things all the time* and *I find it hard to make new friends.* Statements are rated from *strongly agree* to *strongly disagree*, with a resulting total score of 0–50 and higher scores indicating more autistic traits. The AQ has good internal consistency (α = .82; Austin, 2005) and good test–retest reliability (*r* = .70; Baron-Cohen, Wheelwright, Skinner, et al., 2001). AQ scores were significantly higher in the ASD group (*M* = 32.74, *SD* = 9.33) compared with the control group (*M* = 21.91, *SD* = 11.06), *t*(40) = 3.38, *p* = .002, *d* = 1.049, 95% CI for *d* [0.394, 1.693]. Current functioning in the ASD group was assessed using the Autism Diagnostic Observation Schedule (ADOS–G; Lord et al., 2000). Four participants did not meet ADOS criteria for ASD, but they received diagnoses from independent clinicians, they were not outliers on any measure, and their exclusion did not alter the pattern of significance. The study was approved by the University of Surrey Research Ethics Committee.

### Procedure

The order of RMET and MASC administration was counterbalanced across participants. The RMET comprised 36 stimuli depicting the eye section of a face. For each stimulus, participants were required to select one of four verbal labels, presented underneath the image, that best described what the individual was thinking or feeling. Examples include items such as *upset*, *excited*, and *terrified*. The stimuli were identical to those used in the revised RMET (Baron-Cohen, Wheelwright, Hill, et al., 2001) and were presented in black and white at a standard size. Nineteen faces were male and 17 were female. A gender identification control task was not used due to previous ceiling effects in comparable populations (Baron-Cohen, Wheelwright, Hill, et al., 2001). Each stimulus was presented on a computer screen for an unlimited amount of time; however, participants were instructed to respond with their best estimate if they felt unsure of the answer to prevent them from spending too much time looking at each stimulus. Responses were made via a key press. The RMET score was calculated as the total number of correct responses (maximum of 36). The internal consistency of the RMET is modest, with Cronbach’s alpha varying between 0.37 and 0.61 across cultural adaptations (Khorashad et al., 2015; Vellante et al., 2013).

The MASC involved watching a 15-min film depicting four individuals socializing, interrupted at intervals with a mental state or control question about the section of film that had just been played. The 45 mental state questions focused on why characters were behaving in a particular way; it should be noted, however, that 18 of the mental state questions on the MASC are relatively more dependent on emotional mental state decoding (e.g., *What is Betty feeling?*), whereas the remaining questions measure cognitive ToM (e.g., *What is Sandra thinking?*); see Montag et al. (2010). The 21 control questions related to specific details given in the film to ensure that participants were paying attention (e.g., *What time are they meeting?*). Participants responded to each question by selecting from four possible answers and recording their answers on an answer sheet. The MASC score was calculated as the total number of correct responses to the mental state questions (maximum of 45). The mental state questions on the MASC have been shown to have a high internal consistency (α = .84) and high intraclass correlation coefficients (ICCs) in the assessment of test–retest reliability (ICC = 0.97) and have been shown to be sensitive to subtle ToM difficulties in adult participants of normal intelligence (Dziobek et al., 2006). The control question score was calculated as the total number of correct responses to the control questions (maximum of 21; additional control questions were used as per Santiesteban, Bird, Tew, Cioffi, & Banissy, 2015, to provide more possibility for any between-groups variance in control question performance to be detected, if present, and were generated as in the original version of the task, such that they assessed general comprehension and memory for the material but not the understanding of mental states: Control questions are listed in Supplementary Table 1).

## Results

One participant in the ASD group was excluded from MASC analyses due to being an outlier (>3 *SD* from the mean) on this task.

### RMET

**Figure 1 fig1:**
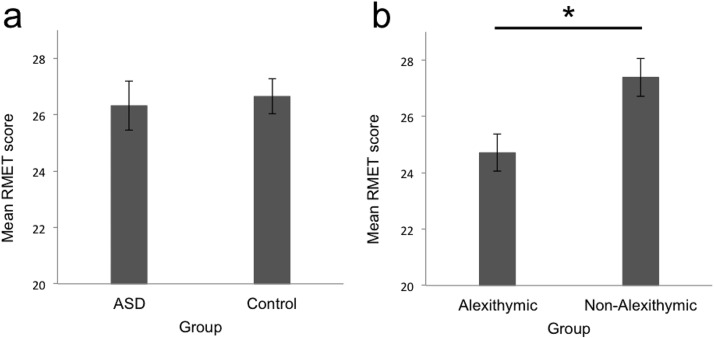
Reading the Mind in the Eyes Test (RMET) performance was unaffected by autism spectrum disorder (ASD; a) but was negatively impacted by alexithymia (b). Error bars indicate standard error of the mean. * *p* < .05.

The ASD (*M* = 26.32, *SD* = 3.77) and alexithymia-matched control (*M* = 26.65, *SD* = 2.99) groups did not differ on RMET score, *t*(40) = 0.32, *p* = .749, *d* = 0.100, 95% CI for *d* [−0.509, 0.707]; Figure 1a. A Bayes factor of 0.317 suggested that the data were over 3.15 times as likely under the null hypothesis as under the alternative hypothesis.[Fig-anchor fig1]

However, when comparing alexithymic and nonalexithymic participants as two groups (regardless of ASD), the alexithymic group (*M* = 24.71, *SD* = 2.84) exhibited significantly worse RMET performance than the nonalexithymic group, *M* = 27.39, *SD* = 3.24; *t*(40) = 2.63, *p* = .012, *d* = 0.861, 95% CI for *d* [−0.187, 1.524]; Figure 1b. A Bayes factor of 4.287 provided strong support for a group difference. The alexithymic group also performed significantly worse than the control participants reported in Baron-Cohen, Wheelwright, Hill, et al. (2001): *N* = 225, *M* = 27.02, *SD* = 3.67; independent samples *t* test: *t*(237) = 2.31, *p* = .021, *d* = 0.638, 95% CI for *d* [0.094, 1.180], illustrating that the alexithymic group was also impaired compared to published normative data on control participant RMET performance.

### MASC

In contrast to the findings for the RMET, the control group (*M* = 35.70, *SD* = 3.31) performed significantly better on the MASC than the ASD group, *M* = 31.22, *SD* = 4.60; *t*(39) = 3.62, *p* = .001, *d* = 1.141, 95% CI for *d* [0.468, 1.801], ruling out the possibility that the ASD group had intact ToM. This significant group difference was supported by a Bayes factor of 36.40. There was no significant difference in the total number of MASC control questions correctly answered between the ASD group and the control group, control *M* = 18.91, *SD* = 1.68; ASD *M* = 18.22, *SD* = 2.39; *t*(39) = 1.09, *p* = .284, *d* = 0.342, 95% CI for *d* [−0.281, 0.961].

No significant difference between alexithymia groups was found for MASC scores, alexithymic *M* = 34.71, *SD* = 4.27; nonalexithymic *M* = 33.22, *SD* = 4.58; *t*(39) = 1.01, *p* = .318, *d* = 0.333, 95% CI for *d* [−0.319, 0.981]. A Bayes factor of .475 indicated that the data were over 2.1 times as likely under the null hypothesis as under the alternative hypothesis.

### Hierarchical Regression Analyses

Hierarchical regression analyses were used to compare the independent contribution of alexithymia and ASD symptom severity to RMET and MASC performance, respectively. These analyses included the effect of gender in the first step, as the gender balance of the ASD and control groups was numerically (although not significantly) different, and gender has been associated with both facial emotion recognition (Forni-Santos & Osório, 2015) and ToM (e.g., Devine & Hughes, 2013). Accordingly, performance on the RMET was analyzed using a hierarchical regression in which gender was entered in the first step, AQ scores were entered in the second step, and TAS–20 scores were entered in the third step. TAS–20 scores remained a significant predictor of RMET performance (standardized β = −.410, *p* = .030, 95% CI for β [−.199, −.011], Δ*R*^2^ = 11.5%), even after accounting for gender and AQ scores, but AQ scores were not a significant predictor in either the second or third steps of the regression (second step: β = −.111, *p* = .490, 95% CI for β [−.124, .061], Δ*R*^2^ = 1.2%; third step: β = .104, *p* = .565, 95% CI for β [−.074, .134]). When analyzing performance on the MASC, gender was entered in the first step, TAS–20 scores were entered in the second step, and AQ scores were entered in the final step. In this model, neither TAS–20 (β = −.161, *p* = .304, 95% CI for β [−.162, .052], Δ*R*^2^ = 2.5%) nor AQ scores (β = −.277, *p* = .125, 95% CI for β [−.243, .031], Δ*R*^2^ = 5.5%) predicted MASC scores; therefore, a follow-up analysis focused on those MASC items that do not ask about feelings and are thus less dependent on emotion understanding, constituting a test of cognitive ToM (Montag et al., 2010). The same analysis applied to these items revealed that AQ scores were a significant predictor of performance (β = −.357, *p* = .046, 95% CI for β [−.188, −.002], Δ*R*^2^ = 9.1%) even after controlling for TAS–20 scores and gender. Full details of the hierarchical regression analyses are presented in Supplementary Table 2.

## Discussion

This study attempted to determine whether the RMET—currently the most popular test of ToM in adults—is a valid measure of ToM or whether performance is in fact determined by emotion recognition ability. Deficits in emotion recognition are commonly associated with alexithymia, and the alexithymia hypothesis of emotion-related impairments in ASD (Bird & Cook, 2013) suggests that in individuals with ASD and comorbid alexithymia, it is alexithymia, rather than ASD per se, that impairs emotion recognition performance. We therefore investigated whether alexithymia or ASD was the better predictor of RMET performance, given the reliance of the RMET on the ability to perceive facial emotion. We also measured whether either ASD or alexithymia could predict performance on a validated ToM task (the MASC), which relies on emotion recognition to a lesser extent than the RMET. Alexithymia, rather than ASD diagnosis, predicted RMET performance, while ASD diagnosis and symptom severity, rather than alexithymia, predicted performance on the MASC task. These results suggest that the RMET may be better characterized as a test of emotion recognition than of mental state understanding.

Should converging evidence for the reliance of the RMET on emotion recognition ability be obtained, substantial revision of conclusions based on over 2,000 studies is required. Such revision will be urgently needed for studies that have been used to support diagnostic or therapeutic approaches with clinical groups (e.g., Anagnostou et al., 2012; Berlim, McGirr, Beaulieu, & Turecki, 2012). In addition, the current data may explain inconsistencies in both the ToM and emotion recognition literatures regarding the abilities of clinical groups. For example, contemporary adaptations of the RMET have failed to show significant differences in performance between ASD and neurotypical participants (Back, Ropar, & Mitchell, 2007; Roeyers, Buysse, Ponnet, & Pichal, 2001), challenging the original RMET findings. The current results suggest that such inconsistent findings are likely a product of sampling variance with respect to alexithymia in ASD samples; those studies with a greater proportion of alexithymic participants in their samples of individuals with ASD would be more likely to report ASD-related deficits than studies with fewer alexithymic participants within the ASD group. Within studies of typical individuals, these results are also likely to explain why poor correlations have been reported between the RMET and alternative tests of ToM, such as the Strange Stories Test and the Faux Pas Test (Ahmed & Stephen Miller, 2011; Spek, Scholte, & van Berckelaer-Onnes, 2010).

The present data suggest that the RMET measures emotion recognition rather than ToM ability. However, it might be argued that the process of emotion recognition could be defined as a form of mental state inference. Under this account, the ability measured by the RMET might be most precisely described as emotion recognition, but the test would still index ToM. The link between ToM and emotion recognition is relatively underinvestigated: Emotion recognition is moderately related to ToM tasks in nonclinical and ASD populations in some samples (Dyck, Ferguson, & Shochet, 2001) but not in others (Bora et al., 2005) and may depend on the particular ToM tasks selected. However, clinical dissociations between emotion recognition and ToM performance lend additional support to the proposal that emotion recognition and mental state inference are discrete cognitive processes. Young adults diagnosed with conduct disorder (especially with callous–unemotional traits) display poor performance on the RMET and other emotion recognition tasks when compared to the typical population (Fairchild, Van Goozen, Calder, Stollery, & Goodyer, 2009; Sharp, 2008). Despite poor emotion recognition performance, the same group tends to perform at an average level on alternative ToM tasks and tends to have intact mentalizing capacity (O’Nions et al., 2014). Conversely, recent research into social functioning in dementia has found the reverse: evidence of impaired ToM with simultaneously intact emotion recognition during a video vignette task (Freedman, Binns, Black, Murphy, & Stuss, 2013). These and similar results would argue against the suggestion that emotion recognition involves the same cognitive processes as ToM.

Our data support the majority of the literature in suggesting that alexithymia is not strongly associated with ToM performance. Moriguchi and colleagues (2006) reported a greater deficit on mentalizing measured via an animated shapes task in a nonclinical sample of individuals with high alexithymia compared to low alexithymia. However, most research contradicts this finding across a number of ToM tasks, including the Strange Stories Test and the false-belief task (Lane, Hsu, Locke, Ritenbaugh, & Stonnington, 2015; Milosavljevic et al., 2016). A recent paper (Gökçen, Frederickson, & Petrides, 2016) demonstrated that although higher TAS–20 scores in a nonclinical sample were correlated with poorer performance on the MASC, alexithymia did not predict ToM performance once autistic traits were controlled for, a finding that is in line with our current results.

One limitation of the current study is that although the control and ASD groups did not differ significantly on alexithymia severity, the ASD group still showed higher alexithymia scores than the control group, with a moderate effect size. Nonetheless, the fact that there was no significant difference in RMET performance between the control and ASD groups, despite a higher mean score for alexithymia in the ASD group, suggests that a more closely matched sample might show even more similar RMET performance than found in our participants. Another limitation that should be considered is the small sample size, particularly with regard to the ASD group. Finally, our sample included a relatively low proportion of participants who met the criterion for severe alexithymia in both the control (33.33%) and ASD (31.58%) groups. Future research could investigate RMET performance in matched groups with higher rates of alexithymia, ideally with an equal proportion of participants scoring above and below the cutoff of ≥61 in each group.

Overall, the present study demonstrates that the RMET indexes emotion recognition rather than ToM ability. It also provides further support for the alexithymia hypothesis of emotion-related impairments in ASD (Bird & Cook, 2013), which posits that, where emotion recognition deficits are observed in ASD, they are attributable to co-occurring alexithymia rather than to ASD per se. We therefore urge caution when using the RMET to measure mental state understanding and encourage researchers to control for alexithymia when using tasks with an emotional component to measure social cognition.

## Supplementary Material

10.1037/abn0000182.supp
