# Supplementary material for: Theory of Mind Is Not Theory of Emotion: A Cautionary Note on the Reading the Mind in the Eyes Test
Source: J Abnorm Psychol. 2016 Aug;125(6):818–23. doi: 10.1037/abn0000182 (PMC4976760; doi:10.1037/abn0000182)
Supplement: Supplementary file 1 [file z2l004152955so1.doc]

**Supplemental Materials**

**'Theory of Mind' is not Theory of Emotion: A Cautionary Note on the Reading the Mind in the Eyes Test**

**by B. F. M. Oakley et al., 2016, *Journal of Abnormal Psychology***

**http://dx.doi.org/10.1037/abn0000182**

**Supplementary Methods**

**MASC Control Questions**

The control questions for the MASC were as used in Santiesteban et al. (2015) and are listed in Supplementary Table 1.

**Supplementary Table 1**

*MASC Control Questions*

| Location in film after ToM question no. | Question text | Answer text | | | | Correct |
| --- | --- | --- | --- | --- | --- | --- |
|  |  | A | B | C | D |  |
| 3 | Who did Betty say Sandra thought was cute? | Pete | Cliff | Michael | David | B |
| 6 | How many times has Sandra met Cliff before now? | Never | Once | Twice | Many times | B |
| 6 | What time are they meeting? | 7pm | 8pm | 9pm | 7:30pm | B |
| 8 | What did Cliff say *wasn’t* a reason for coming? | Drinks | The meal | To flirt | To get the money back from Michael | C |
| 9 | What did Cliff ask for? | A cola | Water | Orange juice | Beer | A |
| 12 | How long were Cliff and his ex together? | 4 years | 3 years | 2 years | 4 months | A |
| 12 | What country did Sandra’s ex go to? | The Netherlands | Sweden | Denmark | Germany | B |
| 13 | How did Cliff likely shave in Sweden? | Outdoors with an electric shaver | As usual, in the bathroom | With a razor and cold water | In his hotel room | C |
| 14 | Who has owned pets? | Michael and Betty | Michael and Cliff | Cliff and Sandra | Betty and Cliff | C |
| 20 | Which character *hasn’t* met two of the other characters before this point? | Sandra | Betty | Cliff | Michael | B |
| 26 | Who does Sandra look at during the toast? | Betty | Cliff | Michael | Paulie | B |
| 29 | Apart from Sandra, who else enjoys motorcycles? | Betty | Cliff | Michael | Paulie | A |
| 30 | How many cups of cream go in the recipe? | 1 | 2 | 3 | 4 | B |
| 33 | What kind of pasta sauce are the four characters preparing? | A sauce with sardines | A sauce with ground meat | A sauce with red peppers | A sauce with salmon | C |
| 37 | How many characters *don’t* drink alcohol? | 2 | 1 | 0 | 3 | C |
| 38 | Which chips does Betty have to play? | The white chips | She can pick any color | The black chips | The same chips that Cliff played | A |
| 39 | How many people is the game intended for? | 1 | 3 | 5 | 4 or 2 | D |
| 44 | Who is likely to have drunk more alcohol by the time they go to sleep? | Michael | Cliff | Sandra | Betty | D |
| 45 | What are Cliff’s favourite leisure time activities? | Playing sports | Engaging in various cultural activities | Going to parties | Reading books | B |
| 45 | Which of the four characters is involved in a relationship? | None of them | Cliff and Sandra | Michael | Betty | A |
| 45 | What was the weather like on that evening? | Cold and dry | Mild and overcast | Rainy | Cold and snowy | A |

**Supplementary Results**

**Supplementary Table 2**

*Hierarchical Regression Analyses*

| **a** |  | **RMET performance** | | | |
| --- | --- | --- | --- | --- | --- |
| **Step** | **Predictor** | ***Β*** | ***p*** | ***R*^2^** | **Δ*R*^2^** |
| 1 | Gender | -.104 | .511 | 1.1% | 1.1% |
| 2 | Gender | -.094 | .556 | 2.3% | 1.2% |
|  | ASD severity | -.111 | .490 |  |  |
| 3 | Gender | -.027 | .864 | 13.8% | 11.5% |
|  | ASD severity | .104 | .565 |  |  |
|  | Alexithymia | -.410 | .030 |  |  |
| **b** |  | **MASC mental state performance** | | | |
| **Step** | **Predictor** | ***Β*** | ***p*** | ***R*^2^** | **Δ*R*^2^** |
| 1 | Gender | .320 | .041 | 10.2% | 10.2% |
| 2 | Gender | .353 | .028 | 12.7% | 2.5% |
|  | Alexithymia | -.161 | .304 |  |  |
| 3 | Gender | .346 | .029 | 18.2% | 5.5% |
|  | Alexithymia | -.012 | .949 |  |  |
|  | ASD severity | -.277 | .125 |  |  |
| **c** |  | **MASC cognitive ToM performance** | | | |
| **Step** | **Predictor** | ***Β*** | ***p*** | ***R*^2^** | **Δ*R*^2^** |
| 1 | Gender | .347 | .026 | 12.0% | 12.0% |
| 2 | Gender | .363 | .025 | 12.6% | 0.6% |
|  | Alexithymia | -.077 | .621 |  |  |
| 3 | Gender | .353 | .023 | 21.7% | 9.1% |
|  | Alexithymia | .116 | .513 |  |  |
|  | ASD severity | -.357 | .046 |  |  |

*Note.* Regression analyses predicting (a) RMET performance (including gender in the first step, ASD symptom severity in the second step, and alexithymia in the third step); (b) performance on all mental state MASC questions (including gender in the first step, alexithymia in the second step, and ASD symptom severity in the third step); and (c) performance on pure cognitive ToM MASC questions (including gender in the first step, alexithymia in the second step, and ASD symptom severity in the third step).
